# Risk Factors Leading to Preference for Extreme Facial Retouching

**DOI:** 10.1089/cyber.2019.0545

**Published:** 2020-01-21

**Authors:** Tamami Nakano, Yusuke Uesugi

**Affiliations:** ^1^ Graduate School of Frontiers Bioscience, Osaka University, Osaka, Japan.; ^2^ Faculty of Medicine, Osaka University, Osaka, Japan.; ^3^ PRESTO, Japan Science Technology, Saitama, Japan.

**Keywords:** face attractiveness, autism-spectrum disorders, self-esteem, uncanny valley

## Abstract

Young women posting their edited face photographs on social networking sites have become a popular phenomenon, but an excessively retouched face image sometimes gives a strange impression to its viewers. This study investigates what personal characteristics facilitate a bias toward an excessively edited face image. Thirty young Asian women evaluated the attractiveness and naturalness of their face images, which were edited in eight different levels—from mild to excessive—by expanding their eyes and thinning their chin. The mildly retouched face was evaluated as more attractive than the original face, but the excessively retouched face was evaluated as unattractive and unnatural in comparison with the original face. The preferred face edit level was higher for one's own face than for others. Moreover, participants with higher autism-spectrum quotient (AQ) scores were found to regard excessively edited face images as more attractive. The attention to detail subscale of the AQ showed a significant positive correlation with the preferred face edit level. The imagination subscale, on the contrary, showed a significant negative correlation with the preferred face edit level. The pupil response for self-face images was significantly larger than those for others' face images, but this difference decreased with higher AQ scores. This study suggests that an increased attractiveness in their mildly retouched face promotes this behavior of retouching one's own face, but autistic traits, which are insensitive to the creepiness of the excessively retouched face, might pose a potential risk to inducing retouch dependence.

## Introduction

Since ancient times, women have been applying makeup to their faces to make themselves look more attractive—they do this by emphasizing their eyes or coloring their lips red, among other methods. In recent times, owing to the development of many convenient and easy-to-use photo-editing software technologies, it is quite popular, especially among young women, to upload edited photographs of their own face on social networking sites (SNS).^1^ Their motive for using SNS is mainly to present themselves online and to compare themselves with others.^2,3^ Thus, the retouched face is a manifestation of their preferred self-images to be seen by an imagined audience. A slightly retouched face may make a good impression on others.^3^ However, excessively retouched faces do not resemble a natural human face, and rather induce a strange impression to many. Nonetheless, we often see excessively retouched face photographs on the SNS. Previous studies have investigated the effect of exposure to modified face and body images on young women,^3–5^ but it remains unclear as to the psychological mechanisms driving facial retouch dependence. This study attempts to expand upon previous research and elucidate which psychological factor(s) act as a bias toward preferring excessively retouched faces in young women.

Photo editing has been shown to have a significant impact on the self-worth of an individual. For example, exposure to manipulated social media photographs of peers decreases body image acceptability and increases self-dissatisfaction in young women.^4,5^ We speculate that low self-satisfaction ultimately leads to a psychological demand to change their image, resulting in a dependence on extreme facial modifications. Self-esteem, the subjective evaluation of one's own worth, is an adequate psychological index for assessing the level of general self-satisfaction.^6^ Facial makeup, which is similar to facial retouching, may have a similar effect of increasing self-esteem in women.^7^ Thus, this study hypothesized that people with low self-esteem prefer stronger facial retouching (Hypothesis 1).

When facial retouching becomes excessive, the human-likeness and naturalness of facial image are lost, and creepiness overwhelms attractiveness. This extreme dependence on facial retouching should be suppressed because the aim of facial retouching is to make a face appear more attractive. However, when the sensitivity to the creepiness of extreme facial retouching is weak, the retouch dependence might not be suppressed. Thus, we focused on autism-spectrum disorder (ASD), defined as a deficit in social communication skills along with repetitive and restricted behaviors and interests.^8^ Studies have shown that individuals with ASD exhibit an impairment in facial recognition, including facial identity recognition and facial expression recognition.^9–12^ They are also insensitive to the creepiness of the extremely manipulated human face and the human-like synthesized voice.^13,14^ This study, therefore, hypothesized that people with higher autistic tendency might prefer stronger facial retouching (Hypothesis 2).

In addition, self-presentation on SNS is an action that is intended at receiving praise by others. Young women who have a higher self-comparison tendency are more negatively affected by exposure to manipulated images.^3^ Moreover, they tend to make social comparisons with their peers more than models and celebrities for both social and physical attributes.^15,16^ This motivation to look better than their peers serves as a bias for preferring stronger manipulation on their face than those of their peers. Therefore, this study hypothesized that young women prefer stronger facial retouching on their own face compared with their friends or unknown peers (Hypothesis 3).

**FIG. 1.**
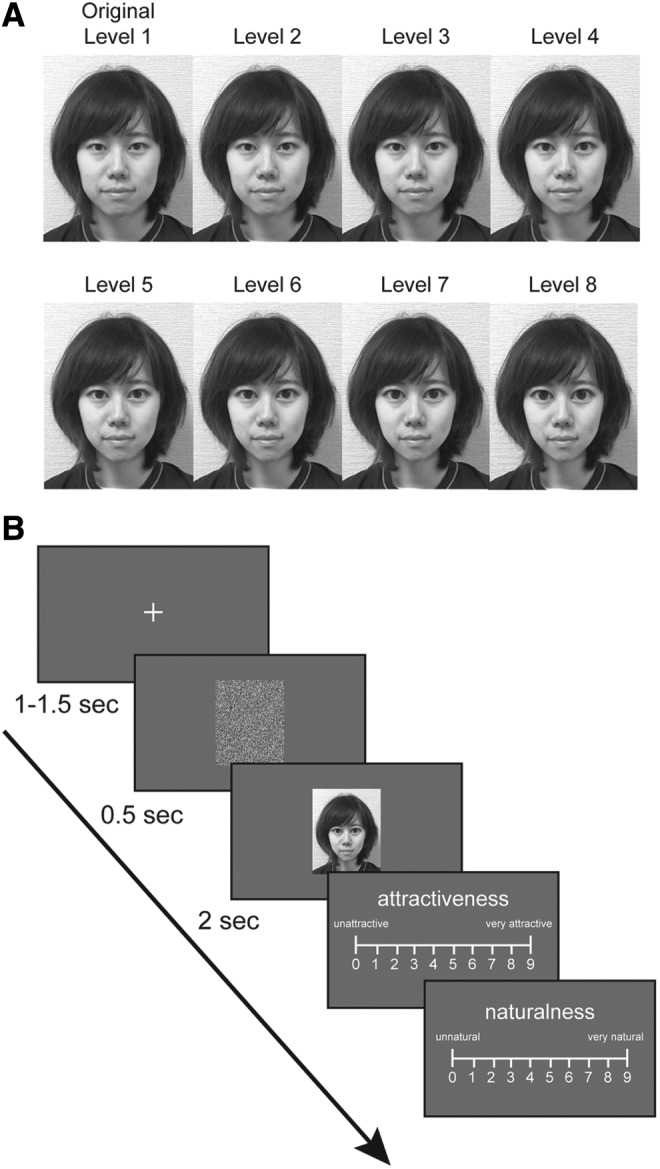
Experimental stimuli and procedures. **(A)** An example of a face image used in this study. Taking the original photograph as level 1, we created seven edited face images with the size of eyes 5.5% larger and the size of chin 1.5% thinner than the previous level for each face. The informed consent to publish the picture was obtained from the model. **(B)** Protocol of the face evaluation task. After watching the face stimuli, participants evaluated the attractiveness and naturalness of the face by inputting a number on the keypad.

To test these three hypotheses, this study examined whether lower self-esteem and/or higher autistic tendency acts as a bias for preferring stronger facial retouch, especially on one's own face. This study was conducted only in young women because retouching behavior is popular in young women, and women have been shown to be more vulnerable to body image-related influences than men.^17^ To identify each individual's most preferred degree of the edited face, we first photographed young Asian women recruited from four social groups in Osaka University and edited their face images—that is, the photographs of their face—to make their eyes look bigger and chin thinner (which is the most popularly used retouch in Asia) at eight different levels (Fig. 1A). Based on their ratings of attractiveness for the various edited images of familiar, unknown, and their own faces, the most preferred degree of edit was identified for each person in each face type. In addition to subjective evaluations, interest in facial images was objectively assessed by measuring pupil size during the experiment. This was utilized because pupil diameter changes unconsciously under the control of the autonomic nervous system and becomes larger when interest or attention to an object is high.^18,19^ Therefore, we examined whether there was a difference in pupil response depending on the face edit level.

## Materials and Methods

### Participants

Thirty female college students with no psychiatric disorders participated in this study (mean age: 21.7, range: 19–31). We recruited them from four social groups; two school clubs (eight participants from each club) and two courses of their graduate school (seven participants from each course). By pairing the two social groups, the same set of photographs were able to be evaluated by both the inner and outer social groups. All reported having normal or corrected-to-normal vision. One participant was excluded from the pupil response analysis because the data were not saved due to a technical glitch. The review board from Osaka University approved this experimental protocol (FBS30-4), and our procedures followed guidelines outlined by the Declaration of Helsinki. All participants provided written informed consent before the experiment.

### Stimuli

Before the experiment, we photographed the faces of all participants to use them as the experimental stimuli. Participants were photographed in their usual face style. All photographs were converted to grayscale and resized to 400 × 500 pixels. They were then edited to make their eyes look bigger and chin thinner using the popular face image editor “SNOW” (Fig. 1A). We created two groups of 15 people, all of whom belonged to one of the two distinct social groups, and asked them to rate a total of 15 faces: own, friends, and others. Thus, the total number of face images used was 120 (15 women × 8 levels). A visual mask (400 × 500 pixels) for each face was created by random shuffling of the face images.

### Apparatus and procedure

The participants were asked to sit on a chair that stabilized their heads on a chin rest to view stimuli presented on a 24-inch liquid crystal monitor (1,920 × 1,080 pixels, 60 Hz; FlexScan, Eizo, Japan) at a distance of 62 cm (46° × 27°). The experiment was controlled by MATLAB software (MathWorks) with Psychophysics Toolbox Version 3. During the experiment, their pupil sizes were recorded using an infrared eye tracker *Tobii Pro Spectrum* (Tobii AB, Sweden) with a temporal resolution of 120 Hz. A five-point method implemented in the software was used for calibration.

At the beginning of each trial, the participants were directed to fixate on a cross (0.5° × 0.5°) that appeared at the center of the monitor for a randomly determined duration between 1.0 and 1.5 seconds (Fig. 1B). Following the visual mask (10° × 12.5°) that appeared at the center of the monitor for 0.5 seconds, a face image (10° × 12.5°) appeared for 2 seconds. Subsequently, the instruction and the scale appeared for participants to evaluate attractiveness of the face on a scale from 0 to 9. After waiting for input of the number, another instruction and the scale appeared to evaluate naturalness of the face on a scale from 0 to 9. The participants input the number using the numeric keypad. The intertrial interval was 1.5 seconds. The session consisted of 120 trials, and the presentation order of the face stimuli was randomized across participants.

After the experiment, the participants filled out three questionnaires. The first one showed a list of all participants' original face images, and they answered whether each face was a familiar face, unknown face, or self-face. The second one was the Japanese version of autism-spectrum quotient (AQ) test, which is a 50-item self-report questionnaire assessing the autistic traits across five subscales (i.e., social skills, communication, attention to detail, attention switching, and imagination) in general populations.^20^ The scale of the AQ ranges from 0 to 50. Eighty percent of those diagnosed with ASD score a ≥32. The crosscultural stability of the AQ test encompasses the Japanese version.^21,22^ The third questionnaire, the Japanese version of Rosenberg Self-Esteem Scale (RSES) test, was a 10-item self-report questionnaire assessing self-esteem related to overall feeling of self-worth or self-acceptance.^23^ Each item is answered on a four-point Likert scale ranging from strongly agree to strongly disagree. The RSES ranges from 0 to 30, where a score <15 suggests low self-esteem.

### Data analysis

Based on the participants' report, average scores of attractiveness and naturalness were calculated for three face groups: familiar face, unknown face, and self-face. The participant's preferred edit level was calculated by multiplying the top three levels by the weight of each score for each face type. The linear mixed model was used for a regression analysis, and model evaluation was based on the Akaike information criteria (AIC) and the Bayesian information criteria (BIC). Lower AIC or BIC value indicates a better model. All data analysis was conducted using Matlab 2017b, and the statistical analysis was conducted using Matlab 2017b and SPSS.

## Results

**FIG. 2.**
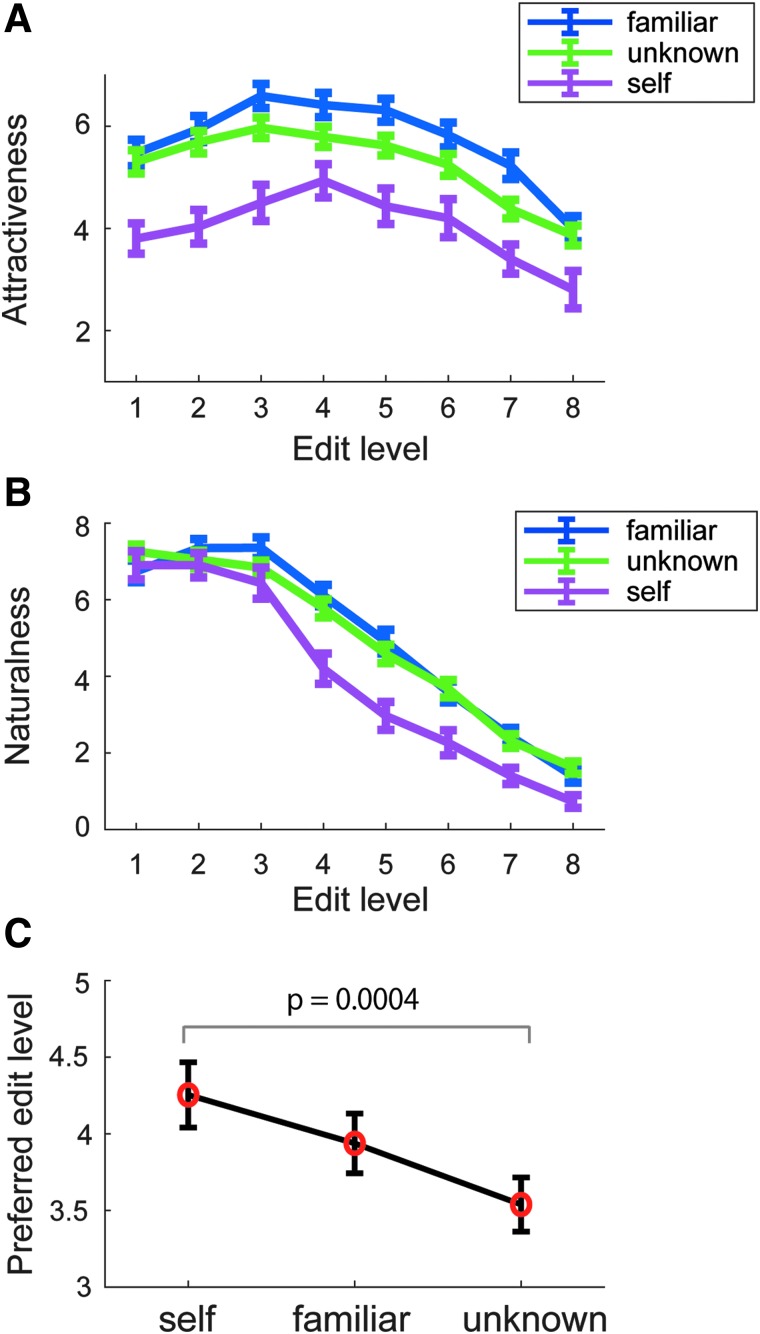
Evaluations for the 8-level face stimuli. The mean ratings of attractiveness **(A)** and naturalness **(B)** for each edit level of the faces across the three face groups (i.e., familiar, unknown, and self-face). **(C)** The comparison of the preferred edit level among self, familiar, and unknown faces. The error bar represents standard error.

First, we compared the impressions of the edited faces for attractiveness and naturalness in each face group—familiar face, unknown face, and self-face (participant's own face). As shown in Figure 2A, the participants found familiar and unknown faces more attractive than their own faces (mean score: familiar face 5.72, unknown face 5.23, and self-face 4.01). The familiar and the unknown faces received the highest ratings of attractiveness at the edit level 3, while the highest rating for attractiveness for self-faces was at level 4. An analysis of variance (ANOVA) with factors of face group (i.e., familiar, unknown, and self-face) and edit level revealed significant main effects, but no significant interaction between them (face group *F* = 27.4, *p* < 0.0001, partial *η*^2^ = 0.49; edit level *F* = 39.1, *p* < 0.0001, partial *η*^2^ = 0.57; interaction *F* = 1.9, *p* = 0.08, partial *η*^2^ = 0.062, *Greenhouse–Geisser correction*). The attractiveness for faces at levels 7 and 8 was significantly lower than that for the faces at levels 1–6, and the attractiveness for faces at levels 3, 4, and 5 was significantly higher than that for faces at level 1 in the Ryan's *post hoc* test with multiple-comparisons correction. The attractiveness for self-faces was significantly lower than that for familiar and unknown faces (self vs. familiar *t* = 7.2, *p* < 0.0001, *r* = 0.28; self vs. unknown *t* = 5.1, *p* < 0.0001, *r* = 0.18).

With regard to naturalness, the participants evaluated that no editing (level 1) for self-face and a slight editing (level 2) for familiar and unknown faces were the most natural (Fig. 2B). As the facial editing progressed, the evaluation score for naturalness dropped dramatically and it fell almost to 0 at level 8. A two-way ANOVA test revealed significant interaction between face group and edit level (*F* = 4.17, *p* < 0.001, partial *η*^2^ = 0.126). In the *post hoc* test with Ryan's multiple-comparisons correction, the scores of naturalness for self-faces were significantly lower than those for familiar or unknown faces across levels 4–8.

We also examined whether the participants' preference of face edit level might be different between self-faces and other faces. As shown in Figure 2C, the preferred face edit level on their own face was the highest, followed by familiar and unknown faces (self 4.25, familiar 3.94, and unknown 3.53). One-way ANOVA revealed a significant main effect of face type on the preferred face edit level (*F* = 7.07, *p* = 0.002, *η*^2^ = 0.20), and the *post hoc* analysis confirmed a significant difference between their own face and the unknown face (*t* = 3.75, *p* = 0.0004, *r* = 0.44).

**FIG. 3.**
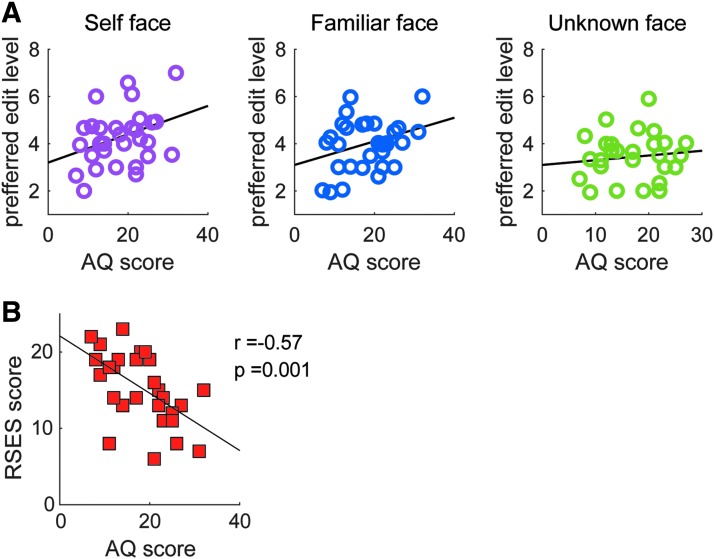
Correlation between the preferred face edit level and personal characteristics. **(A)** Positive correlation between AQ scores and the preferred face edit level in each face group. **(B)** Negative correlation between AQ scores and RSES scores. AQ, autism-spectrum quotient; RSES, Rosenberg Self-Esteem Scale.

Next, we examined whether individual characteristics have an influence on the preferred face edit level. To predict the participants' preferred edit level for the faces, we conducted a regression model that included AQ scores and RSES score as explanatory variables. Model evaluation using AIC and BIC revealed that the model including only AQ scores was better than the model including both AQ and RSES or only RSES (Table 1). As shown in Figure 3A, participants with a higher AQ score preferred a higher degree of facial edit across all face groups (regression slope 0.04, *t*(88) = 2.69, *p* = 0.008, *r* = 0.28). An analysis of covariance (ANCOVA) also confirmed that the main effect of AQ across all face groups was significant (*F* = 7.03, *p* = 0.01, *η*^2^ = 0.08), but the slopes were not significantly different among the groups (*F* = 0.39, *p* = 0.7, *η*^2^ = 0.009). We furthermore conducted a regression analysis using the five subscales of the AQ as explanatory variables. Although the BIC values were lower in the model using the total AQ score, the AIC value was the lowest in the model using the subscales of the AQ. The attention to detail subscale showed a significant positive correlation with the facial edit level (regression slope 0.12, *t*(84) = 2.35, *p* = 0.02), and the imagination subscale showed a significant negative correlation with the facial edit level (regression slope −0.18, *t*(84) = −2.35, *p* = 0.02). The other subscales (i.e., social skill, attention switching, and communication) did not show a significant correlation with facial edit level.

**Table 1.** Summary of the General Linear Models

Model	AIC	BIC	Variable	β	t	df	*p*
AQ	271	**281**	Intercept	3.1	9.3	88	<0.00001
			AQ	**0.04**	2.7	88	**0.009**
Five subscales of AQ	**269**	289	Intercept	3.1	7.3	84	<0.00001
			Social skill	0.05	0.8	84	0.5
			Attention switching	0.05	0.8	84	0.4
			Attention to detail	**0.12**	2.4	84	**0.02**
			Communication	0.13	1.9	84	0.05
			Imagination	**−0.18**	−2.4	84	**0.02**
AQ+RSES	273	285	Intercept	3.6	4.9	87	<0.0001
			AQ	0.04	1.8	87	0.07
			RSES	−0.02	−0.7	87	0.5
RSES	274	284	Intercept	4.7	11.3	88	<0.00001
			RSES	−0.05	−2.0	88	0.05

Bold represents a significant beta value.

AIC, Akaike information criteria; AQ, autism-spectrum quotient; BIC, Bayesian information criteria; RSES, Rosenberg Self-Esteem Scale.

The AQ and RSES scores have high inverse correlation (Fig. 3B, *r* = −0.57, *p* = 0.001). We also examined the correlation between RSES and five subscales of the AQ. The RSES score was significantly correlated with social (*r* = −0.57, *p* = 0.0009), communication (*r* = −0.66, *p* = 0.0001), and attention switching (*r* = −0.56, *p* = 0.0013), but not correlated with attention to detail (*r* = −0.17, *p* = 0.37) and imagination (*r* = −0.29, *p* = 0.12).

**FIG. 4.**
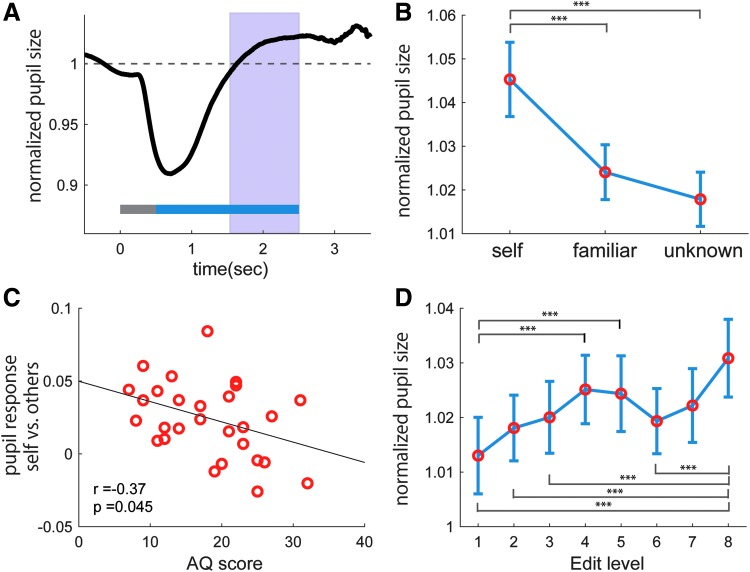
Pupil response to the edited face stimuli. **(A)** Temporal pattern of pupil diameter in response to the visual stimuli. The *gray bar* represents the presentation of the visual mask stimuli, and the *blue bar* represents the presentation of the face stimuli. The *pink area* (1.0–2.0 seconds after the onset of face stimuli) represents the time window of pupil response to the face stimuli. The pupil diameter was normalized using the average value taken at 0.5 seconds before the onset of the visual mask stimulus, which was set as a reference value. **(B)** Comparison of pupil size among the three face groups. **(C)** Negative correlation of the AQ scores with the difference in pupil response between self and others' faces. **(D)** Comparison of pupil size among the different face edit levels. ***p < 0.0001.

We furthermore analyzed objective response for the edited faces by measuring pupil diameter. Figure 4A shows the temporal change in the normalized pupil diameter in response to the visual stimuli averaged across all trials. After pupil contraction due to light reflection, the pupil dilated more than the baseline for 1–2 seconds after the onset of the face stimuli. This time window was used as the pupil response for each face stimulus in the later part of the analysis. The pupil response was significantly larger for self-faces as compared with familiar and unknown faces (Fig. 4B, main effect of face group: *F* = 22.9, *p* < 0.0001, *η*^2^ = 0.45; the *post hoc* test: self >familiar, *t*(56) = 4.99, *p* < 0.0001, *r* = 0.56; self>unknown, *t*(56) = 6.45, *p* < 0.0001, *r* = 0.65). Furthermore, people with lower AQ scores showed larger pupil response toward their own face against others' faces (Fig. 4C, *r* = −0.37, *p* = 0.045).

The pupil response was also different depending on the level of facial edit (Fig. 4D). The level 8 face edit induced the maximum pupil dilation (mean ± standard deviation: 1.031 ± 0.038), and the second peak was observed at edit level 4 (1.025 ± 0.033). In contrast, the original face (level 1) induced the minimum pupil dilation (1.013 ± 0.037). An ANOVA test confirmed a significant main effect of the level of facial edit on the pupil size (*F* = 5.29, *p* < 0.0001, *η*^2^ = 0.16). The *post hoc* test revealed that the pupil response at level 8 was significantly higher than those at levels 1, 2, 3, and 6, and the pupil response at levels 4 and 5 was significantly higher than that at level 1.

## Discussion

This study reveals that even if an edited face image looks unnatural, women regard it as attractive, considering the emphasized eyes on the retouched face. Interestingly, this tendency was especially high in the case of their own faces. External appearance plays a key role in social interactions with others. Women apply facial makeup to increase their facial attractiveness, and it also increases the global self-esteem.^7^ A previous brain imaging study reported that the orbitofrontal cortex, a region known to be involved in representing stimulus-reward value, responded to facial attractiveness,^24^ and a face with makeup induced greater activation in this brain region than the same face without makeup.^25^ Moreover, the ventral tegmental area, which is the center of reward system, showed greater activation for self-face than for unknown face.^26^ These facts suggest that the increased attractiveness resulting from digital editing of the face acts as a reward and motivates people to change the appearance of their face in a more beautiful manner, which is why the participants might have preferred a higher degree of edit for their own face than others.

However, this study also shows that excessively retouched faces lose both naturalness and attractiveness, and are regarded less attractive than a real face. Excessive manipulation of facial features can make a face appear less realistic and evoke a feeling of eeriness, coupled with reduced attractiveness.^27^ The decline in attractiveness associated with excessive facial retouching might be related to the “uncanny valley” phenomenon, in which humanoids that look fairly close to humans but do not fully achieve human-likeness ultimately cause an eerie feeling.^28,29^ It is worth noting that the automatic pupil response across all face edit levels showed two peaks—at level 4 and at level 8. The level 4 face edit was evaluated as the most attractive by the participants, but the level 8 face was evaluated as the most unnatural and the least attractive. These results suggest that not only the face attractiveness but also the face creepiness enhanced visual attention, but the reason they are attracting attention for is actually the opposite. These results indicate that it is important to put a limit on how far one edits their face to the extent that it does not look creepy, to portray a good impression for others on the SNS.

This study furthermore revealed that people with a higher AQ score feel their excessively edited face to be attractive. For example, the participant with the highest AQ score rated the level 7 version of her face as the most attractive, although the other participants evaluated this face image as extremely unnatural and unattractive. Why do people with higher AQ prefer excessively retouched faces? There are several possibilities to explain this result. First, we point out that the AQ score showed a negative correlation with self-esteem. A previous study has demonstrated that social skills have a positive correlation with self-esteem.^30^ Social communication skills are effective in social interactions, and a positive perception of one's social self presumably enhances self-evaluation. On the contrary, people with low self-esteem tend to be dissatisfied with their social self-image, which is why they may have a positive impression on a face that is significantly different from the actual.

However, the AQ score predicted the preferred face edit level more precisely than the score of self-esteem in this study. Thus, other factors besides self-esteem should be correlated to the correlation between AQ and the preferred edit level. We raise a possibility that people with higher autistic trait would rarely find the excessively retouched face to be creepy. Individuals with ASD exhibit an impairment in facial recognition, such as face identity or facial expression processing.^9–11^ Furthermore, the previous study revealed that adults with ASD could not discriminate between human and artificial singing voices, whereas adults without ASD felt the artificial singing voice creepy and unnatural.^13^ Another study has reported that children with ASD did not show the uncanny valley effect toward the face morphed between cartoon and human faces.^14^ These studies indicate that people with higher autistic trait have reduced sensitivity to subtle changes in facial and voice characteristics. It is worth noting that the preferred face edit level was positively correlated with the attention to detail subscale of the AQ. Individuals with ASD exhibited superior local processing along with inferior global processing.^31–33^ This tendency to focus on local facial features may explain why it is more challenging for individuals with ASD to notice the excessive manipulation of facial parts has disrupted the overall facial balance. Based on these facts, we assume that individuals with higher AQ scores preferred the excessively retouched face because they were not too sensitive to the proper balance between emphasizing the features of their face and maintaining the naturalness of the face.

Another important finding is that the automatic pupil response to the self-face was much greater than familiar or unknown faces, but this difference in pupil response between self-face and other faces decreased with the AQ scores. This indicates that people usually have a special interest on their own faces compared with some other face. Previous brain imaging studies have reported that self-face recognition also involves the right-lateralized cortical network, which is not much the case for recognition of other faces.^34–36^ However, the activation in this right-lateralized cortical network for self-face recognition was not observed in ASD individuals.^37^ Given these facts, people particularly give attention to their own face and undergo special information processing related to self-recognition, but people with low social communication skills may not have such a clear separation in recognizing one's own face and another individual's face.

A limitation of this study is the small sample size. This was due in part to the aim of comparing the filter preference between known and unknown peers. The results showed, however, that the filter preference was different between self and peers, not between known and unknown peers. Further study of a larger sample size is needed to compare the filter preference between self and peers. In addition, men have much higher autistic traits than women in normal populations.^20^ Since men also actively post their own faces in SNS, further study is called for to examine whether the present findings are applicable within the male populations.

Adolescence is an important period in constructing one's self-image and establishing ego identity. Anorexia nervosa and body dysmorphic disorder, both of which are related to the disorders of self-recognition, have a higher incidence rate during adolescence than other ages. Since young women are most actively posting their retouched faces on SNS, discrepancies between the retouched self-images on the SNS and the actual self-image in the real world are at high risk of causing self-image distortion. This study finds that, even in young women without psychological disorders, the vulnerability of having higher autistic tendency accompanied by low self-esteem leads to a preference for excessive retouching of self-images. Now that SNS have become a major communication tool, it is necessary to consider means to support young women in constructing appropriate self-images.
